# Leukocyte-associated immunoglobulin-like receptor-1 blockade in combination with programmed death-ligand 1 targeting therapy mediates increased tumour control in mice

**DOI:** 10.1007/s00262-023-03600-6

**Published:** 2024-01-18

**Authors:** Akashdip Singh, Eline T. A. M. Mommers-Elshof, Saskia V. Vijver, J. H. Marco Jansen, Susanne Gonder, Robert Jan Lebbink, Dominique Bihan, Richard W. Farndale, Louis Boon, Solomon Langermann, Jeanette H. W. Leusen, Dallas Flies, Linde Meyaard, M. Ines Pascoal Ramos

**Affiliations:** 1 Centre for Translational Immunology, University Medical Centre Utrecht, Utrecht University, Lundlaan 6, 3584 EA Utrecht, The Netherlands; 2 Oncode Institute, Utrecht, The Netherlands; 3 Department of Biochemistry, University of Cambridge, Cambridge, UK; 4 JJP Biologics, Warsaw, Poland; 5 Nextcure, Beltsville, MD USA

**Keywords:** NC410, Anti-PD-L1, Combination therapy, Collagen, Mouse tumour models

## Abstract

Collagen expression and structure in the tumour microenvironment are associated with tumour development and therapy response. Leukocyte-associated immunoglobulin-like receptor-1 (LAIR-1) is a widely expressed inhibitory collagen receptor. LAIR-2 is a soluble homologue of LAIR-1 that competes for collagen binding. Multiple studies in mice implicate blockade of LAIR-1:collagen interaction in cancer as a promising therapeutic strategy. Here, we investigated the role of LAIR-1 in anti-tumour responses. We show that although LAIR-1 inhibits activation, proliferation, and cytokine production of mouse T cells in vitro*,* tumour outgrowth in LAIR-1-deficient mice did not differ from wild type mice in several in vivo tumour models. Furthermore, treatment with NC410, a LAIR-2-Fc fusion protein, did not result in increased tumour clearance in tested immunocompetent mice, which contrasts with previous data in humanized mouse models. This discrepancy may be explained by our finding that NC410 blocks human LAIR-1:collagen interaction more effectively than mouse LAIR-1:collagen interaction. Despite the lack of therapeutic impact of NC410 monotherapy, mice treated with a combination of NC410 and anti-programmed death-ligand 1 did show reduced tumour burden and increased survival. Using LAIR-1-deficient mice, we showed that this effect seemed to be dependent on the presence of LAIR-1. Taken together, our data demonstrate that the absence of LAIR-1 signalling alone is not sufficient to control tumour growth in multiple immunocompetent mouse models. However, combined targeting of LAIR-1 and PD-L1 results in increased tumour control. Thus, additional targeting of the LAIR-1:collagen pathway with NC410 is a promising approach to treating tumours where conventional immunotherapy is ineffective.

**Supplementary Information:**

The online version contains supplementary material available at 10.1007/s00262-023-03600-6.

## Introduction

The extracellular matrix (ECM) is one of the major components of tumours providing mechanical support, modulation of the microenvironment, and a source of signalling molecules [1]. There is increasing evidence that the ECM plays a crucial role in tumour development and progression, but also strongly impacts response to therapy [2]. Collagen is the main ECM constituent and we have previously shown that blocking the interaction of collagen with leukocyte-associated immunoglobulin-like receptor-1 (LAIR-1) results in tumour control in humanized mouse models [3, 4].

LAIR-1 is an immune inhibitory receptor that is expressed on a wide variety of immune cells, including neutrophils, monocytes and T cells [5–7]. LAIR-1 signalling results in the suppression of several immune cell functions, e.g. dendritic cell activation and differentiation, and NK or T cell cytotoxicity [8, 9]. LAIR-1 binds collagens, including extracellular and transmembrane collagens [10], and proteins containing collagen-like domains, such as surfactant protein D (SP-D), complement component 1q (C1q) and collectin-12 [8, 11–13]. LAIR-1 binding to collagen is dependent on hydroxylation of proline residues by prolyl 4-hydroxylase, which allows for the formation of the collagen triple helix [14]. Other post-translational modifications, such as citrullination, can also influence LAIR-1 binding and signalling [15]. LAIR-1 can also inhibit immune cells through tumour-derived collagens and collagen fragments [14, 16].

Multiple factors can modulate the LAIR-1:collagen interaction. These include soluble forms of LAIR-1, which might be shed from the cell membrane, and LAIR-2, a soluble homologue of LAIR-1 [17]. Mouse and human LAIR-1 have similar affinity for collagen molecules, but mice do not have LAIR-2 [10, 18, 19]. Furthermore, the ECM is regulated differently between species [20]. While certain diseases, including connective tissue disorders or inflammatory disorders, are often associated with disruptions in genes encoding collagens and other ECM components in humans, in mice the disruptions are more commonly found in regulators of ECM production or modification, such as *Bmp4* and *Tgfb1* [21].

The autoimmune-like phenotype of programmed cell death protein 1 (PD-1)-deficient mice suggested a key role for PD-1 in immune regulation, prompting its development for cancer immunotherapy [22, 23]. In contrast to PD-1, unchallenged LAIR-1-deficient mice or mice exposed to colitis or experimental autoimmune encephalitis have no major inflammatory phenotype [24]. However, LAIR-1 deficiency enhances neutrophilic inflammation upon respiratory virus infection [25] and fibrosis in the bleomycin mouse model for scleroderma [26], indicative of an essential role in immune regulation. Additionally, one study showed that LAIR-1-deficient mice present exacerbation of disease in lung cancer models, but that LAIR-1 deficiency does not affect subcutaneous tumour growth [11]. In apparent contrast, we and others have previously shown that treatment with recombinant LAIR-2-Fc fusion proteins, such as NC410*,* inhibits tumour growth in humanized mouse models [3, 27]. NC410 is a LAIR-1 antagonist by binding to collagen with a higher avidity and may have additional effects besides blockade of LAIR-1 signalling [17, 28–30].

While the pre-clinical efficacy of NC410 has been reported, its immunological function remains largely unknown. Here, we studied the impact of LAIR-1 deficiency in murine tumour models and explored the mechanism behind NC410 treatment as monotherapy and in combination with known checkpoint blockers. Moreover, we compared the blocking capacity of NC410 between mice and men. These differences may influence the efficacy of drugs in humans where mouse models were employed during the development.

## Material and methods

### Cell lines

B16-F10 cells (RRID: CVCL_0159) and 2B4 NFAT-GFP reporter lines were cultured in RPMI 1640 (Gibco) supplemented with 10% foetal bovine serum (FBS) (Sigma-Aldrich) and 1% penicillin/streptomycin (P/S) (Gibco) at 37 °C and 5% CO_2_. MC-38 cells (RRID: CVCL_B288) were cultured in DMEM supplemented with 10% FBS and 1% P/S. Cell lines were tested for viral contaminants and genetically validated by short tandem repeat (STR) analysis (IDEXX BioAnalytics) before use in vivo. Cell lines were routinely screened for mycoplasma during culture periods. 2B4 reporter lines expressing hLAIR-1-CD3ζ and mLAIR-1-CD3ζ were described previously [10].

### Mice

Adult (8–21 weeks) C57BL/6N (Charles River, Germany) mice, or *Lair1*^+/+^ or *Lair1*^−/−^ on a C57BL/6N background (Taconic Biosciences) mice were used and maintained in the Central Laboratory Animal Research Facility of the University of Utrecht. MC-38 experiments were all performed in female mice, B16-F10 experiments were performed in male or female mice, as indicated. To generate sufficient mice, *Lair1*^+*/*+^ and *Lair1*^−/−^ mice were offspring of homozygous *Lair1*^+/+^ and *Lair1*^−/−^ breeding pairs. Mice were group housed in individually ventilated cages and acclimatized for at least 6 days after arrival. Mice were kept at a 12:12 dark:light cycle and received food and water ad libitum. Cages were enriched with a shelter and tissue paper.

All experiments were performed in accordance with international guidelines and approved by the local experimental animal welfare body and the Central Authority for Scientific Procedures on Animals. Mice were euthanized by cervical dislocation.

### In vivo tumour models

For the MC-38 experiments, cells were harvested, resuspended in 1:1 Matrigel (Corning)/ Hanks' Balanced Salt Solution (HBSS; Fisher Scientific) and 100,000 cells in 100 µl were injected s.c. in the right flank of the mice. For the B16-F10 experiments, B16-F10 cells were harvested, resuspended in PBS or 1:1 Matrigel/HBSS, and 50,000 cells, unless indicated otherwise, in 100 µl were injected s.c. in the right flank of the mice. Tumour growth was determined 3–5 times per week by calliper measurements (Dasqua), and tumour volume was calculated using the formula $length\times width\times height$. Mice were euthanized when tumour size reached 1500 mm^3^, if tumour tissue showed advanced necrosis or other adverse side-effects were observed. Mice that did not develop tumours throughout the experiment were excluded from figures. In some experiments, blood was collected weekly.

Treatments were prepared at a concentration of 1 mg/ml total antibody in sterile PBS, and 200 µl was administered i.p. twice a week from day 7 onwards. NC410 (human IgG1), anti-PD-L1 (rat IgG2A) and corresponding isotype controls were produced in-house by Nextcure, Inc. Anti-PD-1 (RMP1-14), anti-TGF-β (1D11) and corresponding isotype controls were produced in-house by JJP Biologics. Treatment groups were blinded for researcher measuring tumour outgrowth. Mice were randomly assigned to groups with similar average tumour size, and treatments were assigned to groups randomly using Excel.

### T cell stimulation assay

For T cell stimulation assays, indicated concentrations of anti-CD3ε (145-2C11, BD Biosciences) and 5 µg/ml anti-LAIR-1 (113, Invitrogen) or 5 µg/ml isotype control (eBio299Arm, Invitrogen) were immobilized overnight at 4 °C on U-bottom 96-well plate in 50 µl PBS. Spleens were then collected from wild type mice, and splenocytes were isolated by mechanical dissociation. Pan T cells were isolated from the splenocytes by manual magnetic-activated cell sorting (MACS) using the mouse Pan T Cell Isolation Kit II (Miltenyi Biotec), according to manufacturer’s instructions. Isolated T cells were then washed with PBS and stained with 1 µM CellTrace Violet (Invitrogen) in PBS for 7 min at 37 °C. Staining was quenched with 100% FCS, followed by two washes with RPMI + 10% FCS. 100,000 isolated T cells were then added to previously coated U-bottom 96-well plate and cultured for 3 days at 37 °C with 5% CO_2_.

After 3 days, supernatant and T cells were harvested, washed with FACS buffer (FB; PBS + 0.1% bovine serum albumin + 0.01% sodium azide), stained for surface markers (Supplemental Table 1) and viability dye (Fixable Viability Dye eFluor 780; eBioscience) for 25 min on ice, washed with FB and subsequently measured on LSRFortessa (BD).

### ELISA

Cytokine measurements for mouse granzyme B (R&D Systems #DY1865) and mouse interferon gamma (R&D Systems #DY485-05) were done according to manufacturer’s instructions, after 5 × dilution of harvested supernatants in ELISA diluent for granzyme B measurements, or after 5x (for samples stimulated with 0, 0.5 or 1 μg/ml anti-CD3 stimulation) or 20x (for samples stimulated with 2 or 4 μg/ml anti-CD3 stimulation) dilution in reagent diluent (0.1% BSA, 0.05% Tween 20 in Tris-buffered Saline (20 mM Trizma base, 150 mM NaCl)) for mouse IFN-γ measurements. Optical density at 450 nm and 570 nm, for wavelength correction, was determined on CLARIOstar (BMG Labtech).

### RNA sequencing and analysis

For stimulation, 2.5 μg/ml anti-CD3ε (145-2C11, BD Biosciences) and 2.5 µg/ml anti-LAIR-1 (2C7, in-house) or 2.5 µg/ml isotype control (in-house) were immobilized overnight at 4 °C on U-bottom 96-well plate in 50 µl PBS. Spleens from wild type mice were then isolated, and splenocytes were isolated using manual dissociation. Afterwards, cells were washed twice with FB. Cells were then stained for surface markers under constant agitation at 4 °C, after which cells were washed and resuspended in FB. Approximately 1 million naïve CD8^+^ T cells (TCRβ^+^CD8^+^CD62L^+^CD44^−^) per mouse were then sorted and resuspended at a density of 500,000 cells/ml. 100,000 naïve CD8^+^ T cells were then added to previously coated U-bottom 96-well plate and cultured for 6 h at 37 °C with 5% CO_2_. After 6 h, 200 μl of cells were harvested on ice and lysed in 600 μl TRIzol LS (Invitrogen).

Sequencing was performed at Single Cell Discoveries, a sequencing service provider located in the Netherlands. Total RNA was extracted using the standard TRIzol (Invitrogen) protocol and used for library preparation and sequencing. mRNA was processed as described previously, following an adapted version of the single-cell mRNA sequencing protocol of CEL-seq [31]. In brief, samples were barcoded with CEL-seq primers during reverse transcription and pooled after second strand synthesis. The resulting cDNA was amplified with an overnight in vitro transcription reaction. From this amplified RNA, sequencing libraries were prepared with Illumina Truseq small RNA primers. The DNA library was paired-end sequenced on an Illumina Nextseq™500, high output, with a 1 × 75 bp Illumina kit (R1: 26 cycles, index read: 6 cycles, R2: 60 cycles). Read 1 was used to identify the Illumina library index and CEL-seq sample barcode. Read 2 was aligned to the mouse mm10 reference transcriptome using BWA MEM [32]. Reads that mapped equally well to multiple locations were discarded. Mapping and generation of count tables were done using the MapAndGo script [33].

Differential gene expression analysis was done using DESeq2 (v1.38.3) [34], after which non-protein coding genes were filtered out using biomaRt (v2.54.0) [35]. Gene set enrichment analysis was performed using the clusterProfiler package (v4.6.0) [36]. Transcription (co-)factor analysis was performed using the RegEnrich package (v1.8.0) [37]. All analyses were performed using R (v4.2.2).

### Flow cytometry

For flow cytometry experiments, immune cells from spleen and lymph nodes were isolated using manual dissociation. Tumour tissue was dissociated using the mouse tumour dissociation kit (Miltenyi Biotec), according to manufacturer’s instructions. Single-cell suspensions were prepared, washed with FB, before staining for surface markers for 25 min on ice. Cells were again washed with FB and acquired on LSRFortessa. For intracellular staining, cells were fixed and permeabilized after surface staining using the FoxP3 Staining Buffer Set (eBioscience), according to manufacturer’s instructions, before washing with FB, staining with intracellular markers for 25 min on ice, washing, and acquisition on LSRFortessa.

### Immunofluorescence

Slides from snap frozen tumours were fixed with 4% paraformaldehyde and blocked with Mouse BD Fc Block (BD #553142). Slides were then incubated with anti-collagen I (SouthernBiotech #1310–01) in PBS + 1% BSA for 1 h at room temperature (RT). After thorough washing with PBS, the slides were incubated with secondary donkey anti-goat IgG-AF488 (Invitrogen #A11055) diluted in PBS + 1% BSA for 30 min at RT. Slides were then stained with DAPI for 10 min at RT, washed and finally mounted with VectaMount Permanent Mounting Medium (Vector Laboratories) and Lack@home top coat nail polish (Finess Cosmetics), and allowed to settle before image acquisition on a Zeiss fluorescence microscope (Zeiss) using Zen software and ImageJ.

### 2B4 NFAT-GFP reporter assay

Overlapping synthetic peptides (Toolkits) for human collagen II and III and human LAIR-1-CD3ζ 2B4-NFAT-GFP reporter cell experiments were published previously [38]. Mouse LAIR-1-CD3ζ 2B4-NFAT-GFP reporter cell experiments were performed identically.

For NC410:collagen blocking experiments, Maxisorp 96-well plate (Thermofisher #442404) were coated with 5 µg/ml human collagen I (Sigma-Aldrich #C7774, lot #SLBV1411), 5 µg/ml mouse collagen I (Bio-Rad #2150–1425, lot #152452) in PBS by incubating for 3 h at 37 °C. Plates were washed with PBS twice and, if applicable, pre-incubated with indicated concentrations of NC410 (Nextcure lot #0027-NP045-045-1), or isotype Fc control (Nextcure lot #0027-NP097-102-1) in culture medium by spinning down for 5 min at 250 × *g* at RT and incubating for 2.5 h at 37 °C. 2B4-NFAT-GFP reporter cells were harvested by resuspending and seeded at 1 × 10^6^ cells/ml in 50 µl/well into the medium with fusion proteins and centrifuged for 3 min at 320×*g* at RT. Plates were incubated overnight at 37 °C with 5% CO_2_. The next day, cells were resuspended in FB and GFP expression was measured on a FACSCanto II (BD).

### Statistics

Data were plotted and analysed using GraphPad Prism (v9.3), R (v4.2.2) or FlowJo (v10.8.1). For in vivo experiments, sample sizes were determined a priori using G*power software (v3.1) with *α* = 0.05, power (1 − *β*) = 0.80 and equal group allocation sizes. Significance was determined by linear mixed-effects model, log-rank test, or Kruskal–Wallis one-way ANOVA or a two-way ANOVA followed by multiple comparison as applicable. *P* values below 0.05 were considered significant, and *p* values below 0.1 are indicated in each graph.

## Results

### LAIR-1 triggering inhibits T cell effector functions

**Fig. 1:**
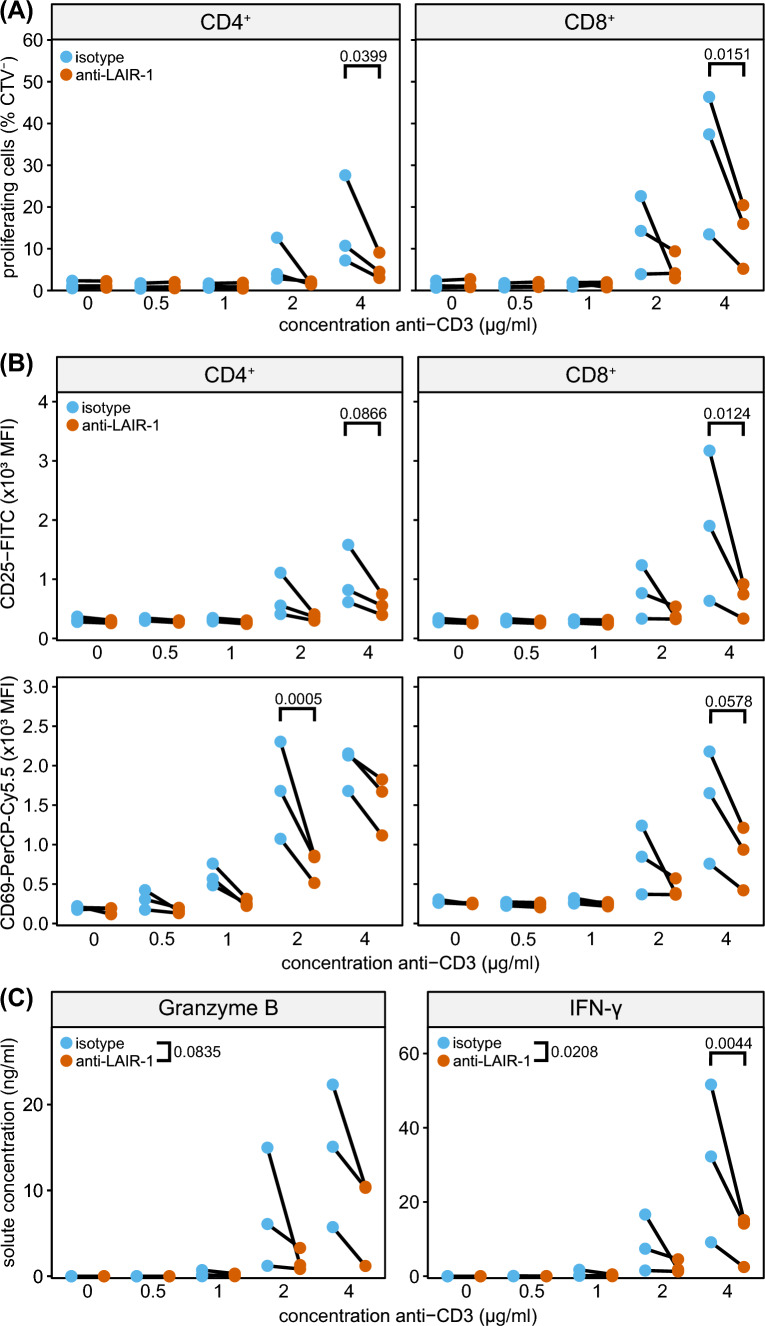
LAIR-1 triggering inhibits T cell effector functions. **A** Pan T cells were isolated from spleen and stimulated with indicated concentrations of immobilized anti-CD3 (clone 145-2C11), in the presence of an isotype control (blue) or an agonistic LAIR-1 antibody (clone 113, red) and proliferation was assessed after 3 days by flow cytometry for both CD4^+^ (left) and CD8^+^ (right) T cells. **B** As in **(A)**, but activation status of T cells, as determined by CD25 and CD69 surface expression, was assessed by flow cytometry. **C** Supernatant of cultures as in **(A)** was harvested and cytokine production of T cell cultures was assessed by ELISA. Graphs depict three mice from one representative experiment. Statistical significance was assessed by two-way ANOVA, for Granzyme B significance is indicated for overall effect of LAIR-1 ligation. *P *values below 0.1 are depicted

To assess the functional role of LAIR-1 in mouse T cells, we performed in vitro T cell stimulation assays. LAIR-1 agonistic antibodies inhibited CD3-induced proliferation of CD4^+^ and CD8^+^ T cells purified from spleen (Fig. 1A). Similarly, anti-LAIR-1 agonistic antibodies reduced CD3-induced CD25 and CD69 expression, as a measure of T cell activation (Fig. 1B). LAIR-1 signalling also decreased granzyme B and IFN-γ production by splenic T cells (Fig. 1C).

To determine which downstream pathways are regulated by LAIR-1, we performed RNA sequencing on naïve CD8^+^ T cells stimulated with CD3 in the presence or absence of anti-

LAIR-1 (Supplemental Figure 1A–B). In line with our in vitro experiments, we observed downregulation of lymphocyte immunity and activation pathways, along with upregulation of adhesion and chemotaxis pathways (Supplemental Figure 1C). We found the involvement of many transcription factors [37], including upregulation of *Lef1,* and downregulation of *Lyar* and *Ybx1* (Supplemental Figure 1D). Thus, ligation of LAIR-1 in vitro results in the suppression of CD3-induced mouse CD4^+^ and CD8^+^ T cell activation and proliferation.

### *LAIR-1-deficiency or blockade does not impact MC-38 and B16-F10 tumour growth *in vivo

**Fig. 2:**
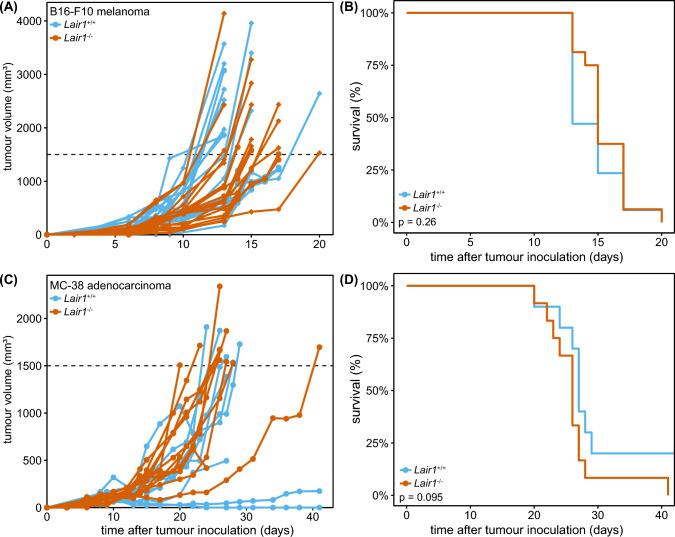
LAIR-1-deficiency or blockade does not impact MC-38 and B16-F10 tumour growth in vivo. **A** 50,000 B16-F10 melanoma cells in Matrigel/HBSS were implanted subcutaneously in the flank of *Lair1*^+*/*+^ and *Lair1*^*−/−*^ mice and tumour growth was assessed by calliper measurements over time. No differences between *Lair1*^+/+^ (blue) and *Lair1*^*−*/−^ mice (red) were observed. Diamonds indicate male mice; circles indicate female mice. Experiment was performed three times, with 6 mice per group. **C** 100,000 MC-38 adenocarcinoma cells in Matrigel/HBSS were implanted subcutaneously in the flank of *Lair1*^+*/*+^ and *Lair1*^*−/−*^ mice and tumour growth was assessed over time. Experiment was performed two times, with 6 mice per group. Two mice were excluded from *Lair1*^+*/*+^ group as they did not develop tumours. Dotted line indicates humane endpoint (HEP: 1500 mm^3^). **B**, **D** Kaplan–Meier curves for experiments performed as indicated in **A**, **C**. Statistical significance was determined by log-rank test

To determine the effect of genetic ablation of LAIR-1 on tumour outgrowth in vivo, we injected *Lair1*^+*/*+^ and *Lair1*^*−/−*^ mice subcutaneously (s.c.) with the B16-F10 melanoma cancer cell line in Matrigel and determined tumour size over time. Neither *Lair1*^+/+^ or *Lair1*^−/−^ mice were able to control tumour growth, and we observed no differences in growth dynamics (Fig. 2A) or overall survival between the two genotypes (Fig. 2B).

As the B16-F10 melanoma is well known for its aggressive growth phenotype [39], we delayed tumour emergence in our model by injecting fewer cells, or injecting tumour cells in PBS instead of Matrigel. Again, we did not observe differences in tumour growth between *Lair1*^+/+^ and *Lair1*^−/−^ mice (Supplemental Figure 2A). The B16-F10 tumours expressed collagen, although the expression in the tumour tissue was low (Supplemental Figure 2B).

Since collagen expression is necessary to impose immune suppression via LAIR-1, we next injected *Lair1*^+/+^ and *Lair1*^−/−^ mice s.c. with the MC-38 colorectal cell line. MC-38 tumours are characterized by a high degree of immune cell exclusion, caused by a dense collagen matrix surrounding the tumour site [40] (Supplemental Figure 2C). Again, tumour growth (Fig. 2C) and overall survival (Fig. 2D) were similar in *Lair1*^+/+^ and *Lair1*^−/−^ mice.

We previously showed that LAIR-1 blockade using NC410, a LAIR-2 Fc fusion protein, decreases tumour outgrowth in a humanized mouse model [3]. Here, we determined whether NC410 also induced tumour control in immunocompetent mice. In contrast to our findings in the humanized mouse model [3], but in agreement with our observations in *Lair1*^−/−^ mice, NC410 treatment intraperitoneally (i.p.) did not result in reduced tumour growth in the s.c. MC-38 tumour model (Fig. 4). Similarly, we found that NC410 single therapy did not result in increased tumour control in the B16-F10 model (Supplemental Figure 3A–C).

**Fig. 3:**
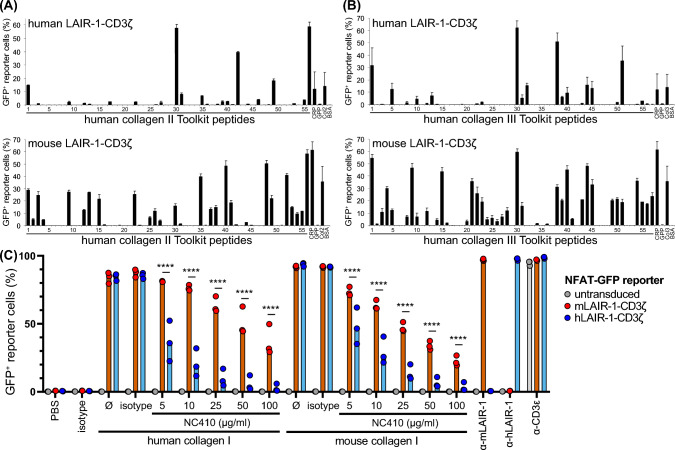
Mouse LAIR-1 signals through a wider array of synthetic collagen peptides and is blocked to a lesser extent than human LAIR-1.** A**, **B** The synthetic peptides of the human collagen II Toolkit (**A**) and collagen III Toolkit (**B**) were immobilized and mouse or human LAIR-1-CD3ζ reporter cells were applied, then LAIR-1 signalling as determined by GFP production was assessed. CRP = Collagen Related Peptide, GPP = peptide consisting of 10 glycine-proline-proline repeats, BSA = bovine serum albumin. Col2 and Col3 indicate immobilized full-length collagen. Human LAIR-1-CD3ζ NFAT-GFP reporter data have been published previously [38]. **C** Human and mouse LAIR-1-CD3ζ NFAT-GFP reporter lines were stimulated with immobilized human and mouse collagen I, and NC410 was titrated to block LAIR-1:collagen binding. Data are aggregated from 3 separately performed experiments with 2–3 technical replicates for each condition. Significance was assessed using a two-way ANOVA

To address this discrepancy, we determined the ability of human and mouse LAIR-1 to bind the synthetic collagen II and III peptide libraries (Toolkits) [38]. We cultured human or mouse LAIR-1-CD3ζ NFAT-GFP reporter cells [10] on plate-immobilized peptides and measured the ability of collagen to induce LAIR-1 signalling by GFP production. We observed that mouse LAIR-1 signalled in response to a larger array of Toolkit peptides than we previously published for human LAIR-1 (Fig. 3A, B). Next, we used NC410 to block human or mouse collagen I-mediated LAIR-1 signalling using the same reporter system and found that NC410 was not able to compete with mouse LAIR-1 to the same extent as to human LAIR-1 (Fig. 3C). Together, our data suggest that mouse LAIR-1 interacts with collagen in a different manner than human LAIR-1 and that LAIR-1 blockade in immunocompetent mice does not impact tumour growth.

### Combination therapy of PD-L1 blockade and NC410 results in increased tumour clearance

Since solely blocking LAIR-1 by NC410 in immunocompetent mice is not sufficient to induce tumour control, additional targeting of other pathways or immune checkpoints might be required. Indeed, NC410, when combined with a programmed death-ligand 1 (PD-L1)

/tumour growth factor beta (TGF-β) blocking reagent, decreased tumour outgrowth in collagen-dense mouse tumour models [4]. Here, we investigated if targeting of either TGF-β or PD-L1 in combination with NC410 increases tumour clearance.

**Fig. 4:**
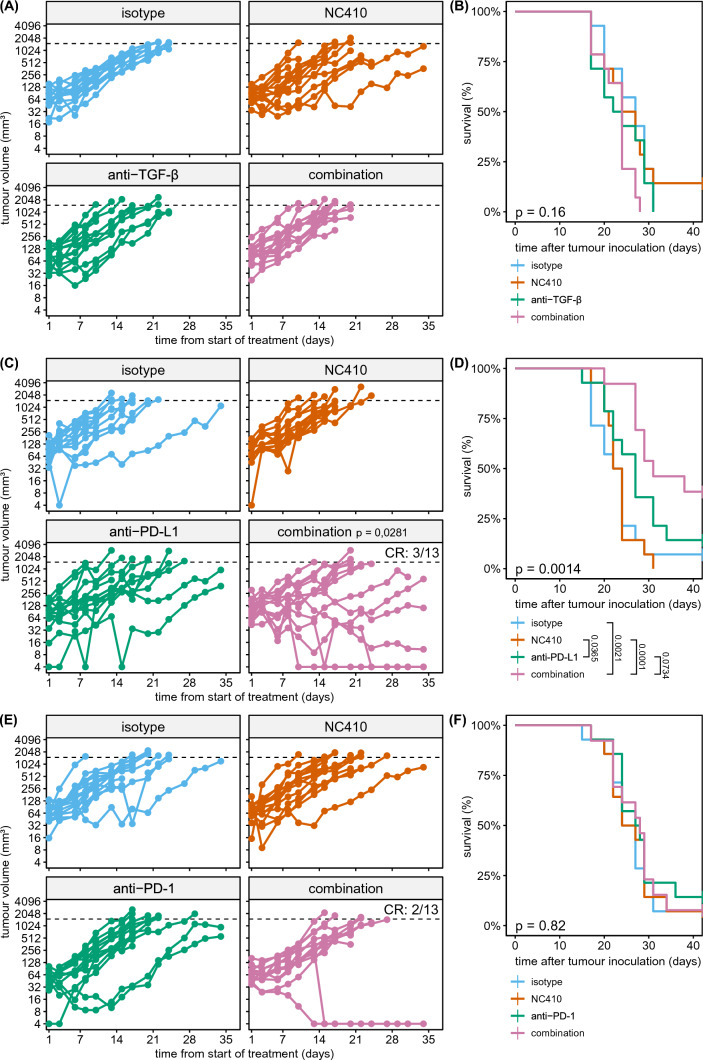
Combination therapy with NC410 and anti-PD-L1 results in decreased tumour burden and increased survival. **A** MC-38-tumour bearing mice were treated with isotype controls (100 µg each) (blue), NC410 (200 µg) (red), anti-TGF-β (200 µg) (green) or a combination of anti-TGF-β and NC410 (100 µg each) (purple) intraperitoneally, twice a week from day 7 after tumour inoculation until the end of the experiment. Tumour outgrowth was determined over time by calliper measurements. **C**,** E** As in **A**, but mice were treated with anti-PD-L1 or anti-PD-1, instead of anti-TGF-β, respectively. Dotted lines indicate HEP at 1500 mm^3^. **B**, **D**, **F** are Kaplan–Meier curves for experiments in **A**, **C**, **E**, respectively. Each treatment experiment was performed once with 14 mice per group. One mouse was excluded from the combination group in **C**, **D**, and one mouse was excluded from the combination group in **E**, **F**, as they did not develop tumours. Statistical significance was determined by linear mixed-effects model for tumour growth (*p < 0.05, compared to isotype) or by log-rank test for survival

NC410 treatment either alone or in combination with anti-TGF-β did not result in differences in tumour growth (Fig. 4A) or survival (Fig. 4B). In contrast, NC410 therapy in combination with PD-L1 blockade demonstrated improved therapeutic activity. While tumour growth was similar in the isotype and NC410 treated groups, mice treated with anti-PD-L1 showed reduced tumour growth (Fig. 4C) and had increased overall survival (Fig. 4D). This was further improved by addition of NC410, as NC410-anti-PD-L1 combination therapy resulted in a complete response in 3 out of 13 mice, and in increased survival compared to isotype or NC410 treated mice (Fig. 4C, D). Of note, NC410 required prolonged and repetitive administration for increased activity in combination with anti-PD-L1 therapy (Supplemental Figure 3D).

Considering that anti-PD-1 and anti-PD-L1 therapies have been reported to have varying degrees of efficacy in the MC-38 model [41, 42], i.e. mediated by differences in Fc engagement [43], we next used anti-PD-1 therapy in combination with NC410 to determine if we could observe a similar synergistic effect. Here, we found no reduction in tumour growth after NC410 or anti-PD-1 single treatment or NC410-anti-PD-1 combination therapy when compared to isotype (Fig. 4E, F).

Overall, these data show that NC410, TGF-β and PD-1 alone or in combination do not demonstrate anti-tumour activity, while NC410 in combination with anti-PD-L1 does reduce tumour burden.

### The NC410 treatment effect appears to require LAIR-1

**Fig. 5:**
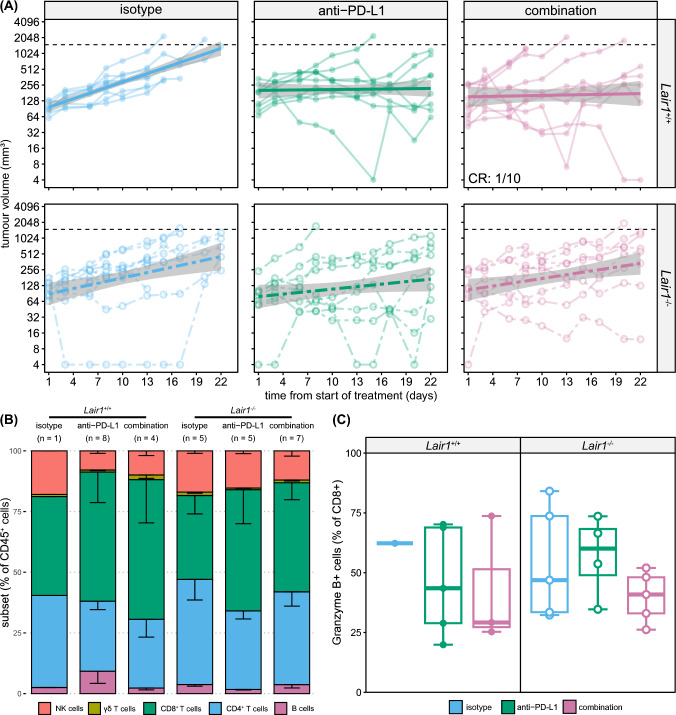
The NC410 treatment effect appears to require LAIR-1. **A** MC-38-tumour bearing *Lair1*^+/+^ (top, solid lines) and *Lair1*^−/−^ (bottom, dotted lines) mice were treated with isotype controls (100 µg each) (blue, left), anti-PD-L1 (200 µg) (green, middle) or a combination of anti-PD-L1 and NC410 (100 µg each) (purple, right) intraperitoneally twice a week from day 7 after tumour inoculation until the end of the experiment. Generalized linear model with standard error (grey) is additionally fitted on top of the data. Tumour outgrowth was determined over time by calliper measurements. Experiment was performed once with 8–10 mice per group. **B** Tumours were collected from mice at sacrifice, and immune infiltrate was assessed by flow cytometry. Relative contribution of NK cells, T cells and B cells was similar across treatment groups. Number of mice per group (*n* = 1–8) is indicated in figure. **C** Tumour infiltrating lymphocytes were assessed for activation based on intracellular granzyme B staining by flow cytometry (*n* = 1–5)

The potential mechanism of action of NC410 is blockade of LAIR-1-collagen interaction [3, 4] (Fig. 3C). To further elucidate this, we injected MC-38 cells s.c. in *Lair1*^+/+^ or *Lair1*^−/−^ mice and treated the mice with isotype control, anti-PD-L1, or a combination of NC410 and anti-PD-L1. Tumour outgrowth in the mice treated with isotype was similar between *Lair1*^+/+^ and *Lair1*^−/−^ mice (Fig. 5A, top- and bottom-left). Although no significant differences were observed, we found that treatment of *Lair1*^+/+^ mice with anti-PD-L1 monotherapy or combination therapy with NC410 resulted in reduced tumour growth compared to isotype, with 1 out of 10 mice demonstrating full clearance after combination treatment (Fig. 5A, top-centre and top-right). Anti-PD-L1 monotherapy resulted in similar tumour control in

*Lair1*^−/−^ and *Lair1*^+/+^ mice (Fig. 5A, top- and bottom-centre). However, addition of NC410 to anti-PD-L1 treatment enhanced tumour control compared to isotype in *Lair1*^+/+^, but not in *Lair1*^−/*−*^ mice (Fig. 5A, bottom-centre and bottom-right). We did not observe differences in immune cell infiltration between the different treatment groups, nor in the activity of the infiltrating CD8^+^ T cells as determined by intracellular cytokine stain for granzyme B (Fig. 5B, C), although a higher degree of CD8^+^ T cell infiltration did correlate with reduced tumour size at sacrifice (Supplemental Figure 4B). Furthermore, we found that the lymphocyte composition between draining and non-draining lymph nodes was similar between treatment groups (Supplemental Figure 4C). Thus, the increased efficacy of NC410 and anti-PD-L1 combination therapy appears to require the presence of LAIR-1 and our data suggest that the main mechanism of action of NC410 is blocking LAIR-1:collagen interaction.

## Discussion

Tumour cells employ many mechanisms to evade the immune system, including modulating the surrounding ECM, contributing to immune cell exclusion and therapy resistance [2, 40]. As collagen, along with other extracellular matrix components, plays a key role in tumour growth and progression [2], it is not surprising that LAIR-1, an inhibitory receptor that recognizes collagen, presents itself as a promising target for cancer immunotherapy in multiple recent studies [3, 4, 27, 44, 45].

In the present study, we observed changes in many transcription factors and regulatory modules after LAIR-1 ligation in the presence of anti-CD3. Amongst them, *Lef1* has been described to play an essential role in maintaining CD8^+^ T cell identity [46], while *Foxo1* is involved in repressing CD8^+^ T cell effector functions [47]. In addition to downregulation of lymphocyte activation pathways, we also observed upregulation of adhesion molecules, such as *Pecam1* and *Sell* (encoding CD62L).

Functionally, we found that LAIR-1 ligation inhibits both mouse CD4^+^ and CD8^+^ T cells after CD3 stimulation in vitro. Therefore, we hypothesized that *Lair1*^−/−^ mice would be able to better control tumour growth. However, this was not the case in both the MC-38 colorectal and the B16-F10 melanoma subcutaneous tumour models. This is in line with previous findings on s.c. B16-F10 tumours in *Lair1*^−/−^ mice [11]. As full knock-out mice have been deficient for LAIR-1 during development, it is possible that there is upregulation of compensatory mechanisms, such as increased reliance on other inhibitory receptors. This should be further explored using inducible knock-out systems or administration of LAIR-1 blocking antibodies.

However, we also found that, in contrast to findings in humanized mouse models [3, 27, 44], treatment with the LAIR-2-Fc protein NC410 did not result in tumour clearance in immunocompetent mice, which aligns with previous data [4]. While mouse and human LAIR-1 have a similar affinity for collagen [10, 18, 19], here we show that mouse LAIR-1 is able to bind to and signal upon a wider array of synthetic collagen-derived Toolkit peptides and that human LAIR-1 signalling is more prone to NC410 blockade than mouse LAIR-1. As the effector cells in the humanized mouse models are human-derived, this may explain some of the discrepancies observed between the mouse and humanized mouse models.

Furthermore, it is important to note that the immune compartment in humanized mouse models is strongly skewed towards T cells and has limited engraftment of myeloid cells [48]. While LAIR-1 is known to be expressed on CD8^+^ infiltrating T cells in the tumour microenvironment (TME) [45], it is also highly expressed on cells of the myeloid lineage, such as monocytes and macrophages [11]. Therefore, it is possible that any benefits from decreased suppression of cytotoxic T cells are offset by increased proliferation or activation of suppressive myeloid subsets, as the presence or absence of these subsets is a major predictor for MC-38 tumour growth dynamics [49]. In agreement with this, a recent study by Keerthivasan et al*.* (2021) showed that genetic ablation of LAIR-1 in the myeloid lineage results in increased tumour burden in a B16-F10 metastasis model [11]. Further studies investigating the effect of NC410 blockade on myeloid cells could resolve this.

Whilst TGF-β blockade was not effective in our experiments, anti-PD-L1 therapy delayed tumour growth and increased survival, and additional treatment with NC410 resulted in further improvement in tumour control. We also found that fewer anti-PD-L1 and NC410 injections resulted in reduced combination therapy efficacy. It is likely that prolonged administration or higher doses of NC410 might be required to maintain effective concentrations at the tumour site, as some NC410 might be lost due to an abundance of collagen in vivo*.*

Interestingly, whereas combination therapy with NC410 and anti-PD-L1 increases tumour control, genetic deletion of LAIR-1 does not further increase the efficacy of anti-PD-L1 treatment. Although NC410 is an effective LAIR-1 antagonist in vitro [3] and is present to a high extent at the tumour site in vivo [4], it is not yet clear whether NC410 only functions by blocking LAIR-1:collagen interactions. It should be noted that NC410 as a fusion of LAIR-2 with a functional IgG domain has additional Fc-mediated functions that could contribute to increased tumour clearance. Furthermore, NC410 also binds with high affinity and avidity to predominant ligands in the TME, including collagen, and may mediate additional effects on immune cells and in the TME through interaction, or compete with other collagen receptors than LAIR-1, as well as mediate direct or indirect effects on collagen remodelling and signalling [3, 4]. LAIR-2-Fc fusion proteins have also been shown to inhibit collagen receptor glycoprotein VI-mediated platelet activation [30], and this could in turn limit disease progression and tumour metastasis [50]. Furthermore, NC410 might also mediate increased tumour clearance through blockade of other ligands than collagen, such as the tumour-promoting complement protein C1q [12, 51]. It is also possible that this is due to immune compensatory mechanisms of *Lair1*^*−/−*^ mice, that are not present during administration of NC410 in *Lair1*^+/+^ mice. Finally, while we observed some differences in tumour growth between isotype and anti-PD-L1 or combination treatment mice, we found no major differences in lymphocyte composition or CD8^+^ T cell activation in the tumours. However, it is still possible that any effects observed on tumour growth are due to other factors, such as differences in the myeloid compartment or the localization of immune cells at the tumour site. To better understand how anti-PD-L1 and NC410 affect tumour growth will require more extensive analysis of the immune infiltrate, including their localization at earlier timepoints.

Taken together, we have found that complete deletion or therapeutic blocking of LAIR-1 with NC410 alone is not sufficient to induce tumour control in immunocompetent mice. We found that combination therapy targeting PD-L1 and LAIR-1 resulted in a reduction of tumour growth, which was not observed in *Lair1*^−/−^ mice in our experimental setting. Importantly, we observed that the therapeutic effect of NC410 differs between pre-clinical models, partly due to species-specific differences in LAIR-1 biology. These findings emphasize the importance of determining differences and similarities in receptor and ligand regulation between species and might assist in selecting the correct humanized or mouse models when targeting specific interactions, such as LAIR-1:collagen. Despite the lack of response to anti-PD-1 in our models, we expect NC410 to complement both PD-1 and PD-L1 targeted therapies in the clinical setting, and NC410 is currently in a phase 2 clinical trial in combination with Pembrolizumab (NCT05572684).

### Supplementary Information

Below is the link to the electronic supplementary material.

Supplementary file1 (PDF 1318 kb)

## Data Availability

RNA sequencing data generated in this study will be made publicly available in Gene Expression Omnibus (GEO) at GSE228709; other data will be made available at request.

## Abbreviations

C1q: Complement component 1q
ECM: Extracellular matrix
LAIR-1: Leukocyte-associated immunoglobulin-like receptor-1
PD-1: Programmed cell death protein 1
PD-L1: Programmed death-ligand 1
SP-D: Surfactant protein D
TGF-β: Tumour growth factor beta
TME: Tumour microenvironment
