# Supplementary material for: Leukocyte-associated immunoglobulin-like receptor-1 blockade in combination with programmed death-ligand 1 targeting therapy mediates increased tumour control in mice
Source: Cancer Immunol Immunother. 2024 Jan 18;73(1):16. doi: 10.1007/s00262-023-03600-6 (PMC10796629; doi:10.1007/s00262-023-03600-6)
Supplement: Supplementary file 1 — Supplementary file1 (PDF 1318 kb) [file 262_2023_3600_MOESM1_ESM.pdf]

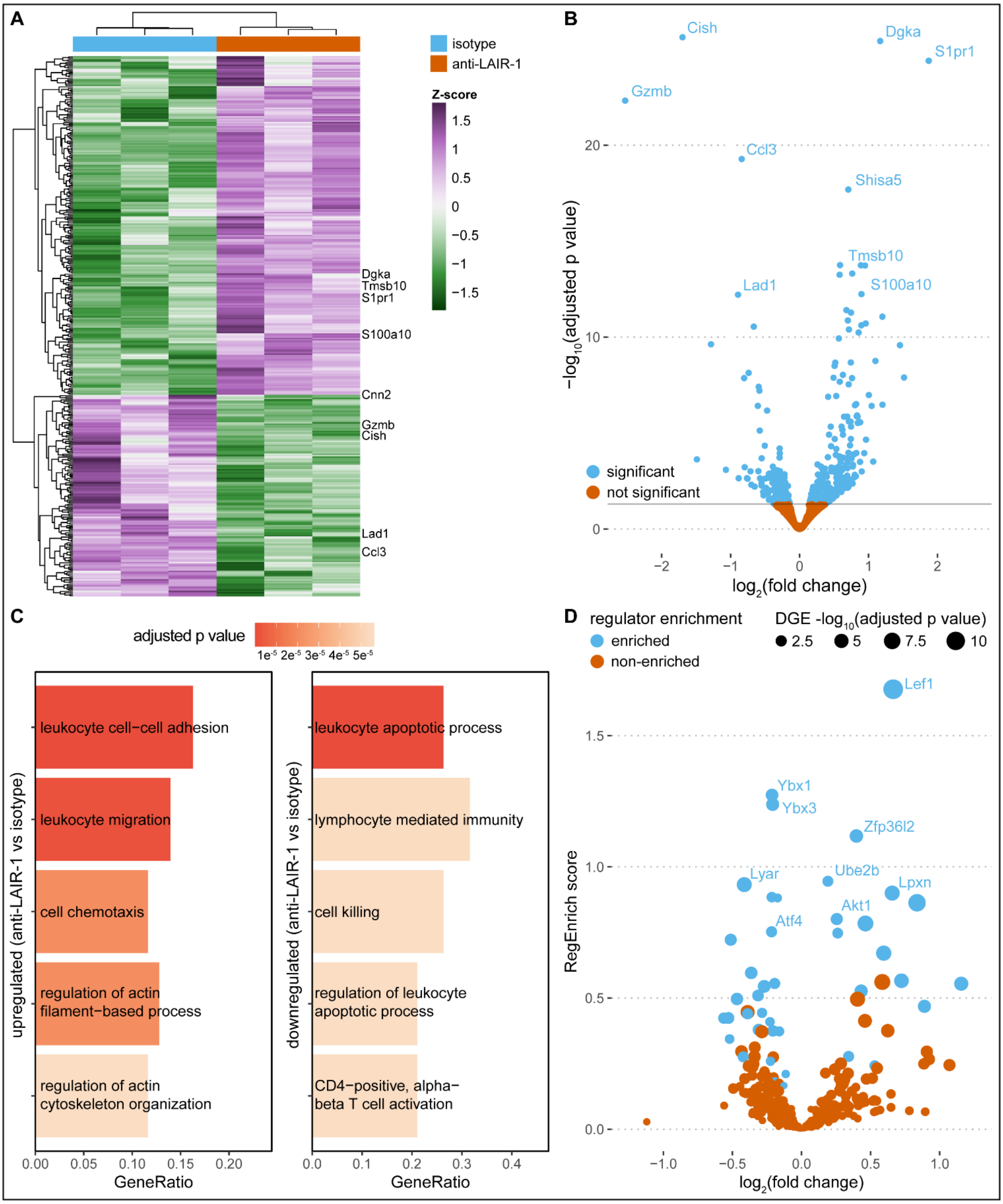

Supplemental Figure 1. LAIR-1 triggering inhibits T cell effector functions. A. Isolated naïve CD8$^+$ T cells from spleen were stimulated with immobilized anti-CD3 (clone 145-2C11), in the presence of an isotype control (red) or an agonistic LAIR-1 antibody (clone anti-LAIR-1(2C7), blue) for 6 hours and RNA sequencing was performed. Row-normalized expression of all differentially expressed genes is depicted. **B**. Volcano plot of log2 fold change in gene expression in the presence of anti-LAIR-1 versus isotype control during 6 hours stimulation with anti-CD3. **C.** Gene ontology pathway enrichment analysis of differentially expressed genes (adjusted p value < 0.05, fold change > 1.5) upregulated (left), or downregulated (right) in the presence of anti-LAIR-1 versus isotype. **D**. RegEnrich score[35] for changes in transcription (co-)factors after stimulation in the presence of anti-LAIR-1 versus isotype control during 6 hours stimulation with anti-CD3. Colours indicate significant regulator enrichment as assessed by gene set enrichment analysis; dot size indicates differential gene expression of corresponding transcription (co-) factors.

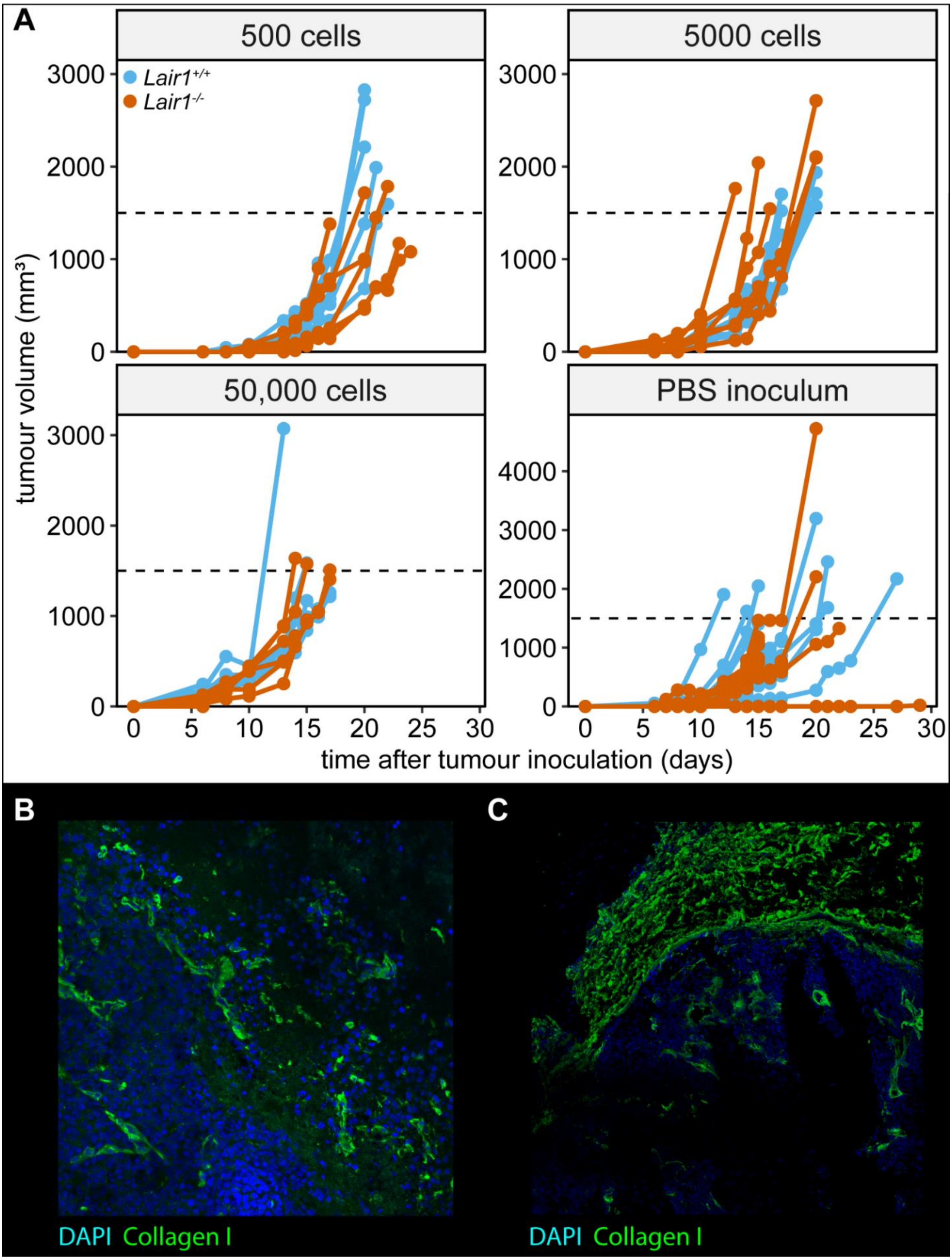

Supplemental Figure 2. LAIR-1-deficiency or blockade does not impact MC-38 and B16-F10 tumour growth *in vivo*. A. Indicated numbers of B16-F10 cells were implanted subcutaneously in the flank of *Lair1*$^{+/+}$ (blue) or *Lair1*$^{-/-}$ (red) mice in Matrigel/HBSS, or 50,000 cells in PBS, as indicated, and tumour growth was measured over time. Titration experiment was performed once, with six mice per group. Data for 50,000 cells also corresponds to Figure 2A. Experiment with PBS was performed twice, with 6 mice per group. **B** and **C.** B16-F10 **(B)** or MC-38 **(C)** tumours were isolated from mice at sacrifice, processed and stained for the presence of collagen I by immunofluorescence. Representative figures for 1 out of 1-3 mice are depicted.

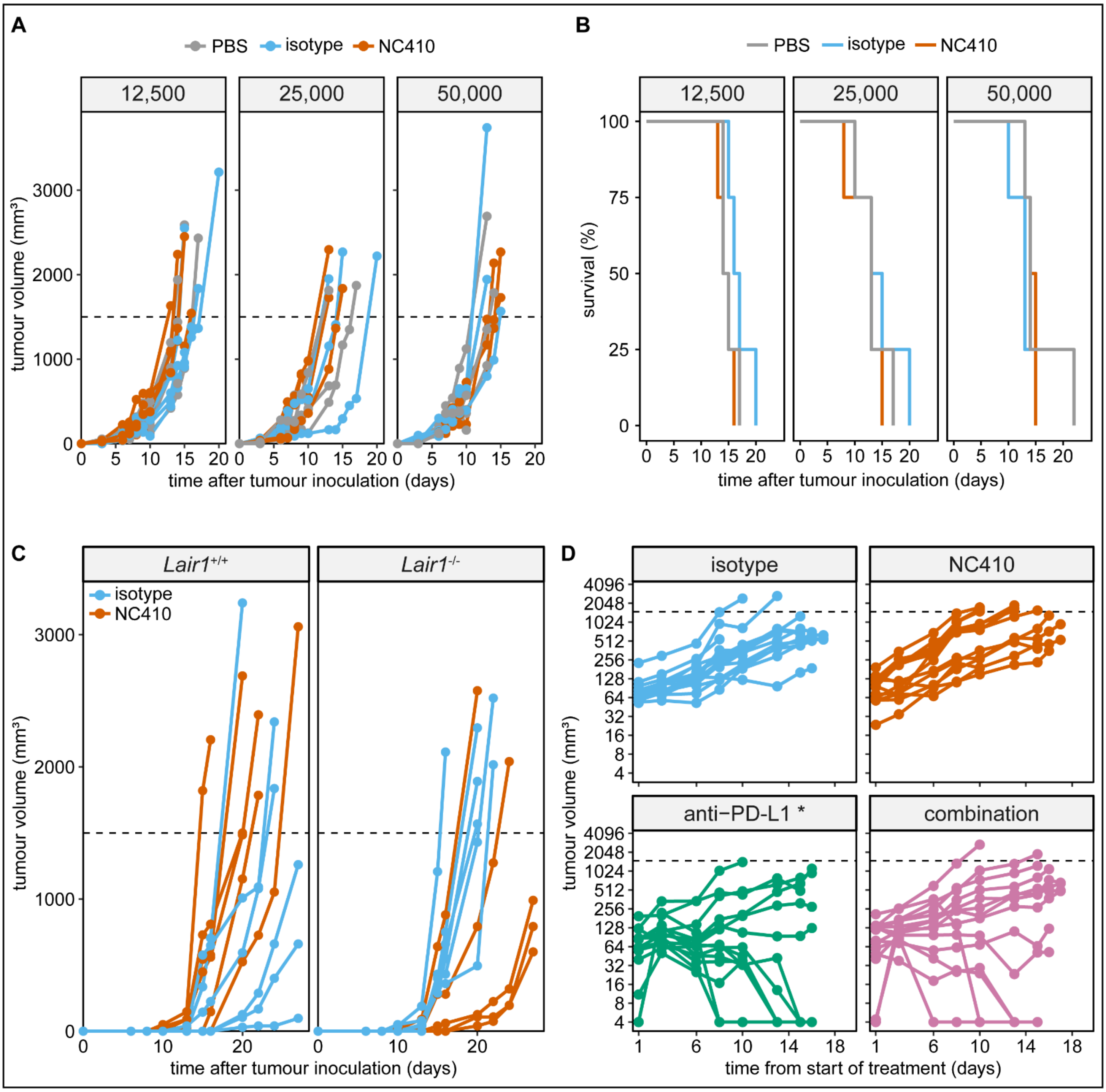

Supplemental Figure 3. Combination therapy with NC410 and anti-PD-L1 results in decreased tumour burden and increased survival. A. Indicated numbers of B16-F10 melanoma cells were implanted s.c. in the flank of wild type mice, and mice were treated i.p. with PBS (200 µl, grey), isotype control (200 µg, blue) or NC410 (200 µg, red) twice weekly. Tumour growth was assessed between treatment groups, but no differences were observed. Experiment was performed once with 4 mice per group. **B.** Survival curves corresponding to **A. C**. *Lair1*$^{+/+}$ (left) and *Lair1*$^{-/-}$ (right) mice were implanted s.c. with 50,000 B16-F10 melanoma cells and treated with isotype (200 µg, blue) or NC410 (200 µg, red) i.p. twice weekly, and tumour growth was assessed. Experiment was performed once with 6 mice per group. One mouse was excluded from the NC410-treated *Lair1*$^{-/-}$ group as it did not develop a tumour. **D.** Wild type mice were implanted s.c. with 100,000 MC-38 tumour cells, and treated three times with isotype controls (100 µg each), NC410 (200 µg), anti-PD-L1 (200 µg) or a combination of NC410 and PD-L1 (100 µg each) on days 7, 10 and 14 after tumour implantation. Tumour growth was assessed until the end of the experiment or HEP. Dotted lines indicate HEP at 1,500 mm^3. Experiment was performed once with 14 mice per group. Statistical significance was determined by linear mixed-effects model for tumour growth (*$p < 0.05$ compared to isotype) or by log-rank test for survival.

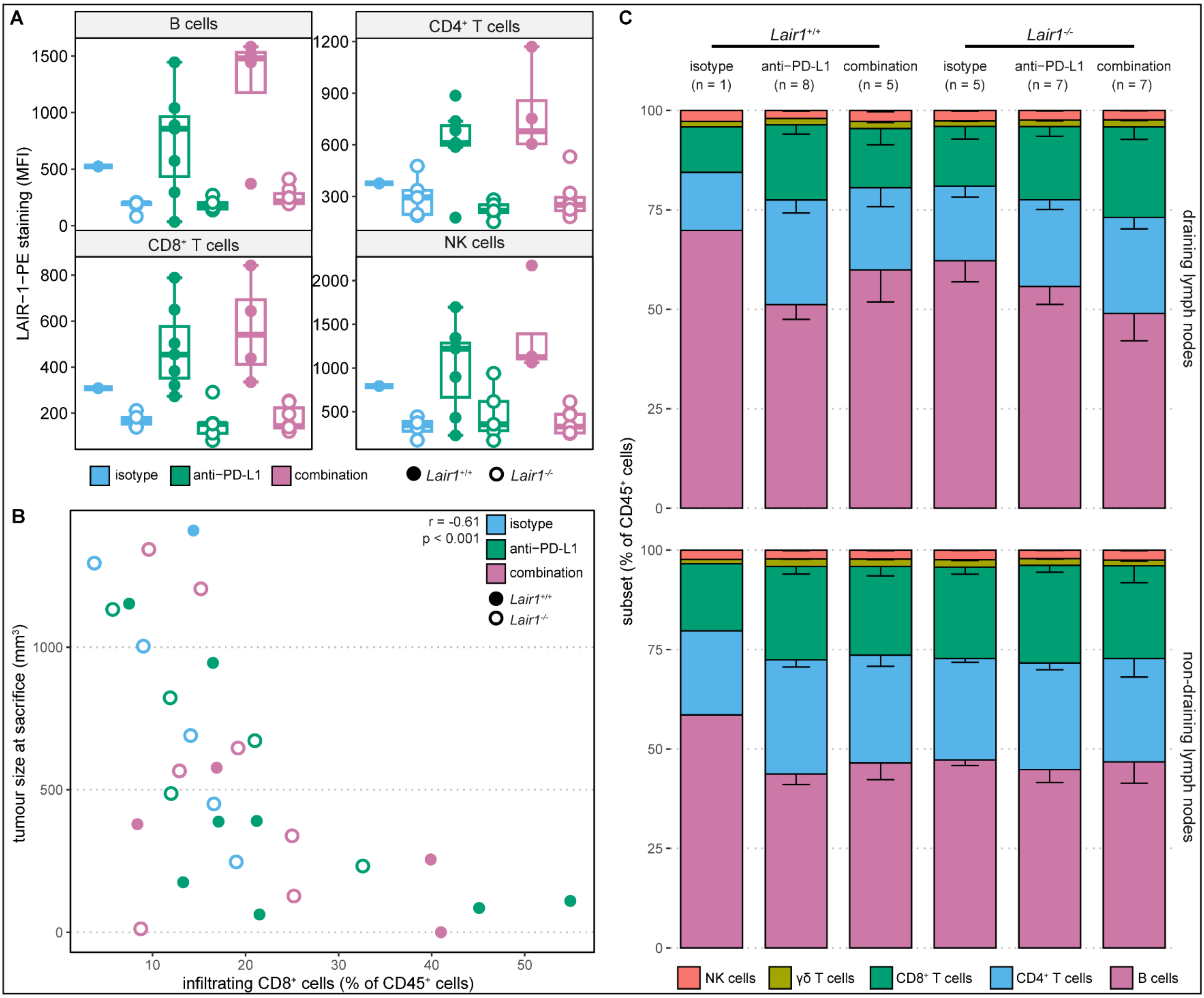

Supplemental Figure 4. The NC410 treatment effect appears to require LAIR-1. A. Tumours were collected from mice at sacrifice, and immune infiltrate was assessed by flow cytometry. The median fluorescence intensity of LAIR-1-PE was determined for B cells, T cells and NK cells (n = 1-8 per treatment group). **B.** Tumours were collected from mice at sacrifice, and immune infiltrate was assessed by flow cytometry. The Pearson's correlation between tumour size at sacrifice was determined as a function of the fraction of infiltrating CD8$^+$ T cells (n = 1-8 per treatment group). **C.** Draining (top) and non-draining (bottom) lymph nodes were collected from mice at sacrifice, and immune infiltrate was assessed by flow cytometry. Percentage of different lymphocyte subsets in CD45$^+$ immune cells are depicted for different treatment groups. Number of mice per group (n = 1-8) is indicated in figure.

Supplementary Table 1. Antibodies used for flow cytometry and immunofluorescence

Target	Fluorophore	Catalog number	Clone	Manufacturer	Dilution used
CD25	FITC	553071	7D4	BD	200
CD69	PerCP-Cy5.5	551113	H1.2F3	BD	100
LAIR-1	PE	12-3051-82	113	eBioscience	600
CD4	PE-CF594	562285	RM4-5	BD	400
CD8a	PE-Cy7	100722	53-6.7	Biolegend	400
	BV421	563898	53-6.7	BD	400
TCR γδ	APC	118116	GL3	Biolegend	100
CD45R/B220	AF700	103232	RA3-6B2	Biolegend	200
CD161 (NK1.1)	PE-Cy5	108716	PK136	Biolegend	100
CD62L	PE-Cy7	25-0621-82	MEL-14	eBioscience	100
TCRβ	BV510	109234	H57-597	Biolegend	200
CD44	BV605	103047	IM7	Biolegend	100
CD45	BV711	103147	30-F11	Biolegend	800
Granzyme B	APC/Fire750	372210	QA16A02	Biolegend	100
Collagen I	Unlabeled	1310-01	Polyclonal	SouthernBiotech	100
Goat-IgG	AF488	A11055	Polyclonal	Invitrogen	1000
